# Lapping of Soft-Brittle Lithium Niobate Crystal with Fixed Abrasive Pad

**DOI:** 10.3390/ma19112299

**Published:** 2026-05-29

**Authors:** Nannan Zhu, Xiaojun Gao, Chao Tang, Jiapeng Chen, Yongwei Zhu

**Affiliations:** 1 Engineering Technique Training Center, Nanjing University of Industry Technology, Nanjing 210023, China; 2 College of Mechanical and Electrical Engineering, Nanjing University of Aeronautics and Astronautics, Nanjing 210016, China; 15150657808@163.com (X.G.); tangchao1@nuaa.edu.cn (C.T.); 3 School of Electro-Mechanical Engineering, Zhongyuan Institute of Science and Technology, Xuchang 461000, China; 15937311032@163.com; 4 Research Center for Advanced Micro-/Nano- Fabrication Materials, School of Chemistry and Chemical Engineering, Shanghai University of Engineering Science, Shanghai 201620, China

**Keywords:** fixed abrasive, lapping, soft and brittle crystal, material removal rate, subsurface damage

## Abstract

Lithium niobate (LiNbO_3_, LN) single crystal is widely used in optoelectronic fields due to its excellent performance. However, its low hardness, high brittleness, and strong anisotropy lead to low processing efficiency and poor surface quality. Hydrophilic fixed abrasive lapping technology was adopted for the thinning of LN wafers in this research. The effects of lapping pressure on the thinning process were investigated comprehensively in terms of the material removal rate (MRR), surface quality, and subsurface damage (SSD). The results show that lapping pressure exerted a significant influence on the machining performance. High pressure contributed to improving the MRR but aggravated surface roughness (Ra) and SSD. With low pressure, material removal was dominated by ductile removal machining, with fine scratches as the main damage form, which was favorable for obtaining low Ra and low SSD. The root mean square (RMS) of the acoustic emission (AE) signal rose with the increase in pressure, increasing slowly in the ductile removal regime and rising abnormally in the brittle removal regime. It was positively correlated with the MRR and SSD and can be used as an in situ monitoring indicator. After a comprehensive comparison of five groups of experiments, 7 kPa was determined to be the optimal lapping pressure, with the following corresponding parameters: wafer speed: 100 rpm; lapping table speed: 80 rpm; slurry flow rate: 100 mL/min; eccentricity: 60 mm; soft lapping pad; abrasive mass fraction: 50%; and lapping time: 5 min. Under these conditions, the Ra value was approximately 30 nm, the MRR exceeded 1 μm/min, and SSD was as low as 3.3 μm, realizing the synergistic optimization of high-efficiency and low-damage machining. It provides a favorable foundation for the subsequent processing of LN substrates, such as ultra-precision polishing, thin-film transfer, and bonding.

## 1. Introduction

Lithium niobate single crystal (LiNbO_3_, LN, Mohs Hardness 5) possesses excellent ferroelectric, piezoelectric, and optoelectronic properties, making it an enduring functional material. At present, it has been applied in infrared detectors, laser modulators, optical switches, frequency doublers, filters, and other devices [1,2,3,4]. High-precision and low-damage machined surfaces are prerequisites for LN-based devices to maintain excellent performance. Therefore, extremely strict requirements are imposed on their machining accuracy and surface quality. Nevertheless, LN crystals feature low hardness, high brittleness, and strong anisotropy, resulting in low efficiency and poor surface quality during processing [5,6]. Attaining high efficiency and ultra-precision machining of LN wafers has become an urgent problem that needs to be solved.

Several studies have been conducted on the thinning and lapping of LN. Wu et al. [7] fabricated LiNbO_3_/Si hybrid wafers using a combination of wafer bonding with a plasma-activated surface and CMP techniques. After the bonding process, the LiNbO_3_/Si hybrid wafers were lapped with boron carbide and aluminum oxide with abrasive slurry sizes of 30 and 10 mm, respectively, and then polished with an amorphous silica with an abrasive slurry size of 32 nm. During the lapping stage, the MRR reached 4.5 μm/min, but the Ra value was as high as 850 nm, and cracks appeared at the edge of the LiNbO_3_ layer. After polishing, the Ra of the LN surface decreased to 1.5 nm, but the MRR was only 0.1 μm/min, indicating relatively low efficiency. Yang et al. [8] introduced a process involving wafer bonding, thinning, and polishing. The thickness of the LN wafer can be reduced to 50 μm. After polishing, the surface roughness can reach 1.63 nm. Although the application of RZJ-304 allowed the bonded and thinned LN wafer and Si wafer to be separated easily without causing any damage to the LN wafer, obvious scratches on the LN surface were still observed. The size of the LN wafer used in the experiment was only 10 mm × 10 mm, the issue of production scale was not resolved, and the processing efficiency was not mentioned in the study. Tian et al. [9] conducted grinding experiments utilizing a high-performance fiber dipped in an HSLP (High-Shear and Low-Pressure) abrasive system composed of a mixture of PEG 200, fumed silica, and 1 μm diamond abrasive grains, to investigate the feasibility of grinding LiNbO_3_ with a flexible ball-end body-armor-like abrasive tool (BBAAT). Under optimal grinding parameters, the Ra decreased from 307.2 nm to 8.2 nm, and the maximum material removal depth (MRD) reached 114.92 μm during the experiments. However, the grinding contact area was limited by the size of the grinding head, and a significant number of diamond abrasive grains were found embedded in the workpiece surface within the grinding area, severely degrading the optical properties of the LN. Huo et al. [10] adopted micro-machining to form LiNbO_3_ crystals with a micro-end milling process using single-crystal diamond tools. A strong crystallographic orientation effect on the surface and edge quality was observed due to the high anisotropy of the material. On the ductile mode machined surface, regular tool marks and less surface defects were observed with an Ra of 10 nm. In contrast, on the brittle mode machined surface, regular tool marks are absent and instead irregular streaks were observed with an Ra of 50–150 nm. Surface defects were found on the micro-machined surface, and edge chipping was observed in most of the slots. Muratov et al. [11] achieved the required surface quality of lithium niobate utilizing the mechanization of the lapping and polishing process and deduced the formula recommended for calculating the total depth of the deformed layer caused by lapping and the minimum polishing time required for its removal. While the lapping tools experienced significant wear, to ensure high productivity, the lap rotation speed (as well as the cutting speed) should be selected as high as possible. Additionally, a deformed layer of material was formed on the surface layer as a result of the impact of abrasive grains. Jeong et al. [12] investigated the CMP (chemical mechanical polishing) process characteristics of LN material. To achieve a higher MRR, additives such as an oxidizer and a chelating agent were incorporated into KOH-based slurry. The highest MRR of 1.2 μm/min was attained with a 2 wt% H_2_O_2_ solution and 0.06 M citric acid in the KOH-based slurry. However, the MRR values were low, resulting in inefficient processing, and the polishing slurry contained environmentally unfriendly components, namely weak acids and strong bases.

Chemical mechanical polishing (CMP) is commonly employed for the surface planarization of materials. Acids, bases, and other chemical substances are often added to the polishing slurry to enhance the material removal rate [13,14]. However, the balance between chemical and mechanical actions is highly dependent on the abrasive concentration [15]. In addition, several defects, such as particle adhesion, slurry residue deposition, scratching, and pitting, occur on the surface [16]. Fixed abrasive polishing (FAP) technology consolidates abrasive grains into the lapping/polishing pad matrix. Only necessary chemical reagents such as oxidizers, wetting agents, and pH adjusters are added in slurry, without abrasive grains contained [17,18,19,20]. Compared with CMP, FAP features small subsurface damage, good workpiece surface uniformity, high processing efficiency, high abrasive utilization rate, and environmental friendliness because abrasive grains are fixed in the pad [21,22], attracting extensive attention from researchers. FAP has also been applied in the lapping and polishing of soft and brittle crystals. Sha et al. developed an integrated approach combining analytical modeling, numerical simulation, and experimental evaluation to investigate crack evolution in the fixed abrasive lapping of zinc sulfide (ZnS, Mohs Hardness 3). The research showed that both crack density and SSD depth increased with applied load. The proposed approach established a practical framework for damage prediction and provided a tool for guiding the machining of brittle materials [23]. Zhang et al. adopted a novel approach of CMP for cadmium zinc telluride (CZT, Mohs Hardness 2.0~2.5) wafer lapping and polishing. Fixed abrasives of SiC grains were used during lapping processes, which was effective in eliminating the embedding of free abrasives [24]. Hang et al. proposed a method of polishing lithium tantalite (LT, Mohs Hardness 5) wafer with fixed abrasive plates to improve processing efficiency. After 30 min of polishing under the optimized parameters (ω = 60 rpm, e = 90 mm) and the application of a variable load, the surface roughness Sa of the workpiece can be reduced to 1.234 nm, and the MRR can reach 0.247 μm/min [25]. Li et al. processed calcium fluoride (CaF_2_, Mohs Hardness 4) crystal via FAP and investigated the effect of FAP characteristics, including abrasive type, particle size, and matrix hardness, on the MRR and surface quality. The better surface quality with Sa of 7.27 nm and an MRR of 192 nm/min was achieved in FAP of CaF_2_ crystal [26].

Although the above studies have achieved certain progress, problems such as low processing efficiency, surface damage, and subsurface damage still remain. To overcome these shortcomings in the ultra-precision machining of LN, this study proposes lapping single-crystal LN with hydrophilic FAP technology. The main purposes of the lapping process were to thin the wafer, reduce the surface roughness, and lower SSD, laying a foundation for subsequent polishing. The MRR in the lapping stage is most significantly affected by lapping pressure. Continuous lapping under high pressure may cause deep indentation of abrasive grains, leaving coarse scratches and new surface/subsurface damage, which requires a longer polishing time to remove. Conversely, low-pressure lapping leads to shallow abrasive indentation and poor pad self-condition (pad surface glazing continually), resulting in low lapping efficiency and high processing costs. Therefore, on account of the special material properties of LN wafers, this research adopted different lapping pressures and optimized the thinning process based on comprehensive experimental results regarding lapping efficiency, surface quality, and subsurface damage layer depth.

## 2. Materials and Methods

### 2.1. Sample Preparation

**Figure 1:**
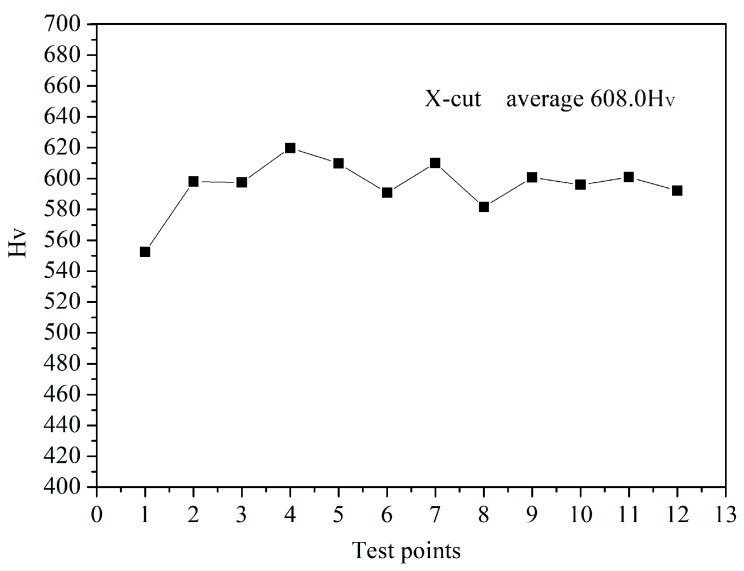
Microhardness results of the X-cut of lithium niobate crystal.

The workpieces in this experiment were X-cut wafers of LN single crystal with a diameter of 70 mm and a thickness of 0.5 mm. Before lapping, the LN blanks had deep cutting textures and poor surface quality (Ra ≈ 230–270 nm). The microhardness measured with a microhardness tester (ZRYST-10000, Zhongyan, Shanghai, China) was approximately 608.0 HV (Figure 1), indicating a low surface hardness. The X-cut crystal plane has two crystallographic directions, namely, the Y-axis and the Z-axis. Nano-scratch experiments confirmed that the critical cutting depth of the X-cut was 77.4 nm along the Y-axis and 36.2 nm along the Z-axis [27], meaning the machining mode transforms from ductile to brittle when the cutting depth reaches 36.2 nm, reflecting high brittleness. Considering the soft and brittle characteristics of LN crystals, a soft lapping pad with W28 diamond abrasives was used in this lapping experiment. The process parameters were optimized to obtain low surface roughness and small subsurface damage layer depth.

### 2.2. Pad Preparation

**Figure 2:**
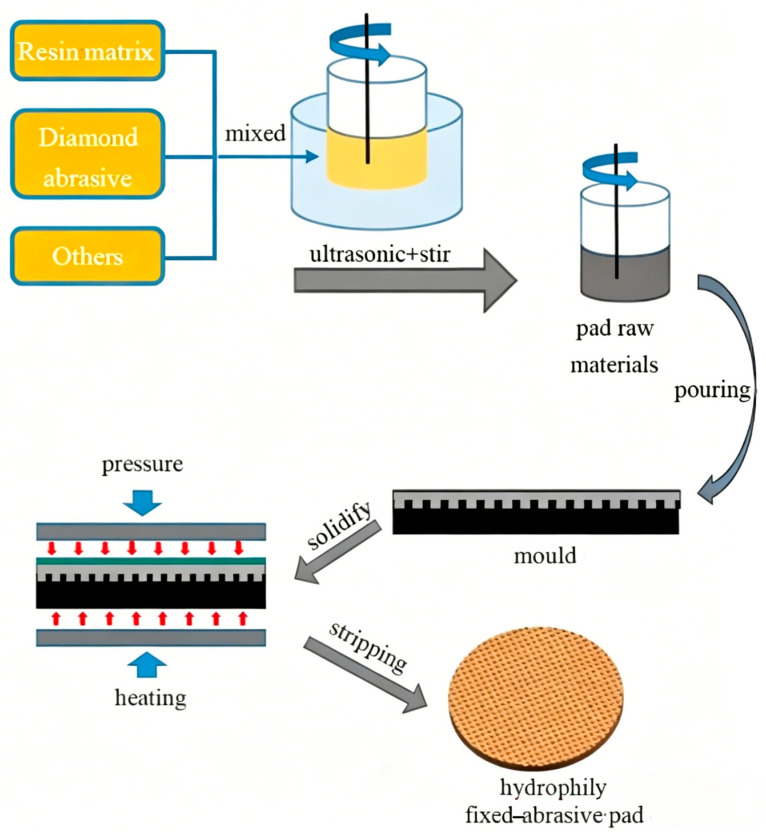
Preparation method of fixed abrasive pad.

A self-made hydrophilic diamond FAP lapping pad was used in this experiment, as shown in Figure 2. First, hydrophilic high polymers were mixed in a certain mass ratio to form a resin matrix, followed by adding abrasives of appropriate type and particle size, additives of proper type and ratio, and a certain mass of accelerator and curing agent. The mixture was ultrasonically stirred to prepare raw materials for the hydrophilic lapping pad, which was then injected into a mold and molded via heating and pressing with a vulcanizing machine (SN-50TY/8, Senna, Wuxi, China). After demolding, the hydrophilic FAP pad was obtained. The abrasive added in the FAP pad preparation was W28 diamond microparticles, and other additives included pore-forming agents such as magnesium sulfate particles. The additives and components are listed in Table 1.

### 2.3. Lapping Experiment Design

**Figure 3:**
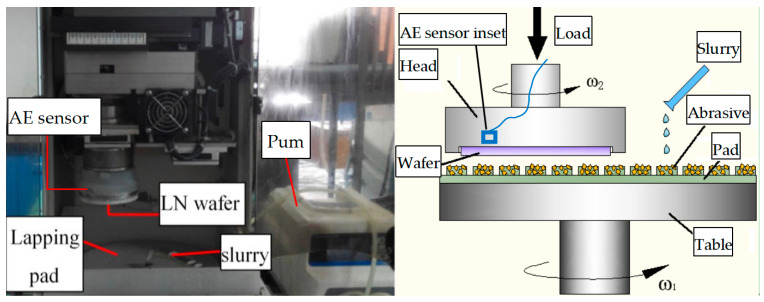
Schematic of lapping principle.

To explore the lapping process of LN wafers with FAP, a group of fixed abrasive lapping experiments were carried out on a friction and wear tester (CP-4, CETR, Campbell, CA, USA). A FAP lapping pad fixed with W28 diamond abrasives was pasted on the workbench. The lapping principle is shown in Figure 3. The lapping time was set to 5 min. The final lapping parameters were determined by taking the MRR, Ra, and SSD as evaluation indicators. The pressure was set to 7 kPa, 10.5 kPa, 12.5 kPa, 15 kPa, and 20 kPa, respectively, with the other factors listed in Table 2. The lapping slurry was a mixture of 1000 mL deionized water and 2 mL OP-10 emulsifier (99%, Dengfeng, Tianjin, China), without additional abrasives or chemical reagents. Before each lapping process, the fixed lapping pad was dressed with an oilstone for 30 s to ensure sufficient exposure of fresh abrasive grains. Each experiment was repeated three times and averaged.

After lapping, the wafers were ultrasonically cleaned and dried for testing. The MRR was calculated according to Equation (1) after the experiment. The Ra values were measured with AFM (CSPM, Benyuan, Guangzhou, China) (probe scanning range 20 μm × 20 μm), and the surface/subsurface micromorphology of the wafers was observed with an optical microscope.

The MRR (μm/min) was calculated as follows:

(1) $$\mathrm{MRR}=\frac{\Delta m\cdot H_{i}}{M_{i}\cdot t}\times 10^{6}$$

where Δ*m* is the mass difference of the wafer before and after lapping (g), *H*_i_ and *M*_i_ are the initial thickness (mm) and mass (g) of the wafer, and *t* is the lapping time (min).

### 2.4. Subsurface Damage Depth Measurement

**Figure 4:**
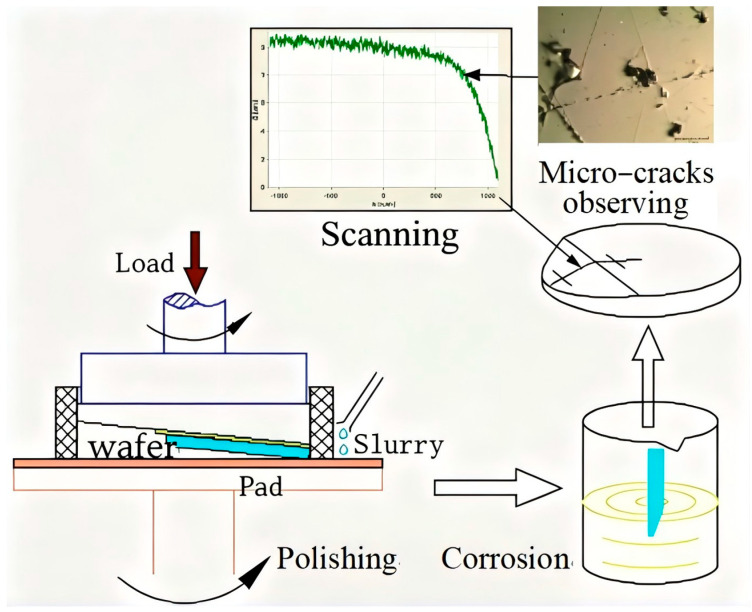
Measurement principle and process of subsurface damage depth.

The subsurface damage depth (SSD) of the lapped LN wafer was detected based on the angle polishing method. Angle polishing tests were carried out on a plane ring polishing machine (Nanopoll-100, Zhibang, Jinhua, China). K9 glass with a small angle (<3°) was selected as the carrier, fixed with an adjustment ring to maintain stable polishing angle. The polishing parameters are listed in Table 3. After polishing, the wafers were unloaded, ultrasonically cleaned, and dried. The inclined surface of each wafer was etched in hydrofluoric acid (40%, Xuxin, Chuzhou, China) solution at room temperature for 30 min to expand cracks and observe easily. Scanning start and end points were marked on the inclined surface, and a 3D profilometer (NanoMap 500LS, AEP Technology, Santa Clara, CA, USA) was used to scan the inclined surface profile as the reference line for SSD measurement. Finally, the wafer was placed on a micro-motion platform, and a metallurgical microscope (MT40, OUMIT, Suzhou, China) was used to observe the distribution and type of cracks on the inclined surface. The SSD was confirmed based on the coordinates of the crack termination position. The measurement principle is shown in Figure 4.

### 2.5. Acoustic Emission Signal

Acoustic emission (AE) refers to the phenomenon that transient elastic waves are generated by the sudden release of strain energy due to deformation or damage inside or on the surface of a material under stress [28,29]. The AE signal data of each group were recorded with a CP-4 friction and wear tester during the experiment, and the root mean square (RMS, V) of the signal voltage within the sampling time was calculated using Equation (2):

(2) $$\mathrm{RMS}=\sqrt{\frac{1}{N}\sum_{i=1}^{N}x_{i}^{2}}$$

where *N* is the number of sampling points within the sampling time, and *x*_i_ is the amplitude corresponding to the *i*-th sampling point.

## 3. Results and Discussion

### 3.1. Experimental Results of MRR and Ra

**Figure 5:**
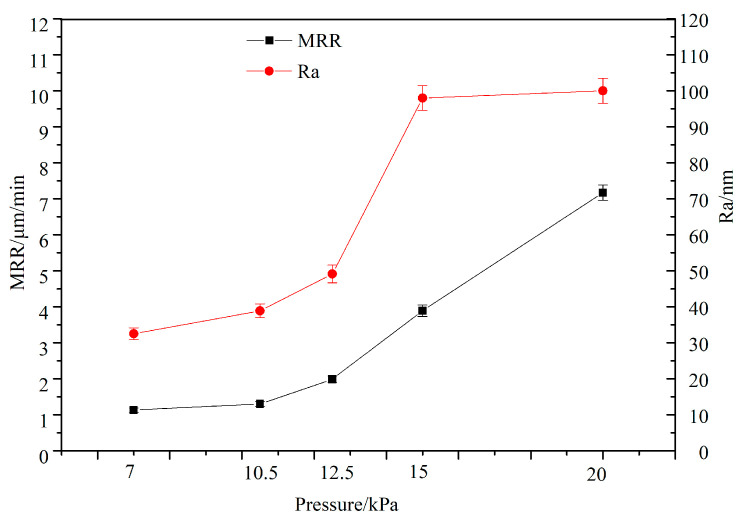
Effect of pressure on MRR and Ra.

**Figure 6:**
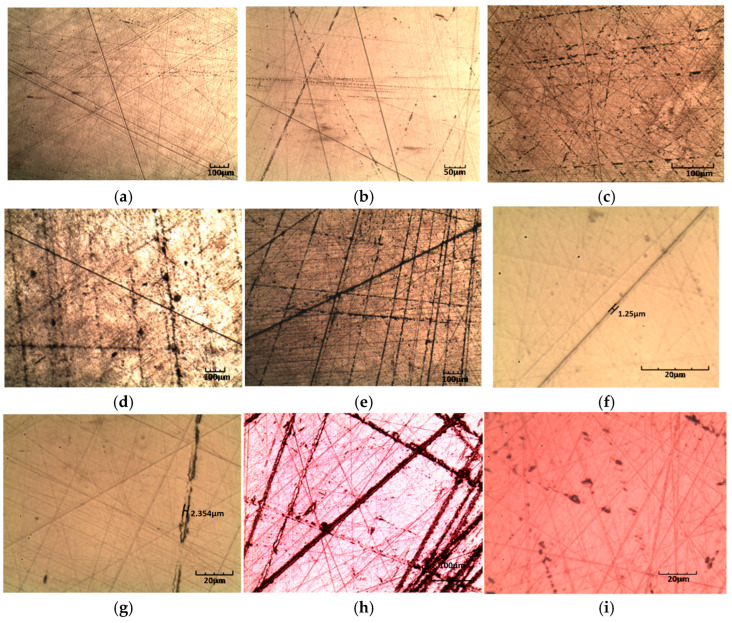
Surface morphology of lapped wafer: (**a**) 7 kPa; (**b**) 10.5 kPa; (**c**) 12.5 kPa; (**d**) 15 kPa; (**e**) 20 kPa; (**f**) fine scratches caused by plowing; (**g**) coarse scratches; (**h**) coarse scratches extend with edge fracture; (**i**) local spalling and pits.

The experimental results of the MRR and Ra for each group are shown in Figure 5. With increased lapping pressure (P), the MRR and Ra of the wafer both showed an upward trend. Ra increased slowly at first with the increase in P, and then increased rapidly when *P* continued to rise, and finally remained basically stable. The surface morphologies of the wafers are shown in Figure 6. The MRR increased slowly at first and then significantly continuously with the increase in P. This result may be attributed to the soft resin matrix of the lapping pad: in the low pressure range, abrasive grains retreated under load, with shallow cutting depth into the LN crystal surface, remaining in the ductile removal dominated by plowing. As shown in Figure 6a–c, when *P* is between 7 and 12.5 kPa, the surface micromorphology distributes mostly fine scratches (Figure 6f). With the increase in P, scratches gradually deepen, and coarse scratches appear at 12.5 kPa (Figure 6g), leading to slow increases in the MRR and Ra. When *P* increased to 15 kPa, the MRR increased rapidly, possibly because the increased pressure enlarged the abrasive cutting depth close to the critical cutting depth of LN, with simultaneous ductile and brittle removal. As shown in Figure 6h, coarse scratches accompanied by edge crack extension and local spalling pits and fractures (brittle removal, Figure 6i) are observed on the wafer surface, resulting in rapid increases in the MRR and Ra. When *P* further increased to 20 kPa, the retreat of abrasive grains intensified. After the abrasive grains on the pad surface became blunt, the plowing friction force increased, leaving coarse sliding scratches on the wafer surface and raising the proportion of brittle removal. Repeated actions caused the blunt abrasive grains to detach from the pad surface. Meanwhile, fresh and sharp cutting edges were gradually exposed. The timely replacement of abrasive grains enabled the MRR to increase rapidly and continuously. Most fresh sharp cutting edges perform plowing (ductile removal), so Ra does not increase significantly, and a large number of coarse scratches can be seen in Figure 6e. From the perspective of material removal, high pressure was beneficial to the MRR; from the perspective of surface quality, low pressure was more conducive to obtaining smooth surfaces.

### 3.2. Experimental Results of Subsurface Damage Depth

**Figure 7:**
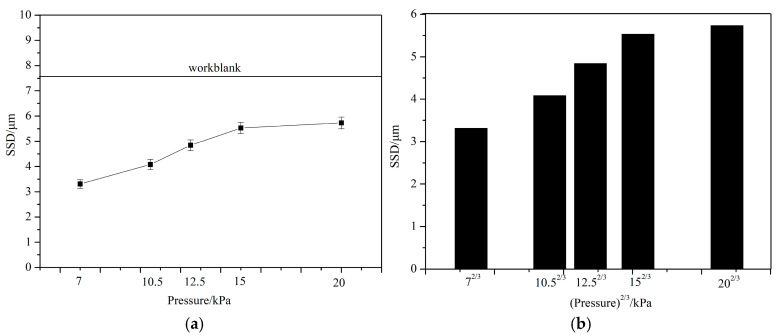
Effect of pressure on subsurface damage depth: (**a**) relationship between SSD and pressure; (**b**) relationship between SSD and (pressure)^2/3^.

The SSD results are shown in Figure 7. All experiments reduced the SSD of the wafer surface and SSD increased with the rise in pressure. When the *P* increased from 15 kPa to 20 kPa, the SSD growth rate was less than 5%. This trend was consistent with that of Ra.

According to indentation fracture mechanics, the theoretical formula (3) for calculating the median crack depth c under sharp indentation shows that the crack depth c generated on the wafer subsurface during lapping is proportional to the applied load P. The higher the load, the deeper the crack depth under the contact zone, (hereinafter referred to as Lambropoulos’ theory) [30,31].

(3) $$c={\alpha_{K}}^{2/3}{\left(\frac{E}{H_{0}}\right)}^{\left(1-m\right)2/3}{\left(\cot\psi \right)}^{4/9}{\left(\frac{P}{K_{r}}\right)}^{2/3}$$

where *c* is the median crack depth (μm), *Ψ* is the indenter angle (°), *P* is the load (Pa), *H*_0_ is the surface hardness of the material (GPa), *E* is the elastic modulus (GPa), *K*_r_ is the plastic stress intensity factor of the indentation stress field, *m* ≈ 1/3–1/2 is a dimensionless constant, and *α*_K_ = 0.027 + 0.090(*m*−1/3).

During fixed abrasive lapping, the limited protrusion height of abrasive grains and significant retreat of abrasive grains caused by the soft resin matrix led to relatively shallow cutting depth into the wafer. Meanwhile, the high concentration of diamond abrasive grains fixed in the resin resulted in a large number of abrasive grains participating in lapping. Under the same load, the force acting on a single abrasive grain was small, so the crack depth was shallower than the SSD of the workblank.

**Figure 8:**
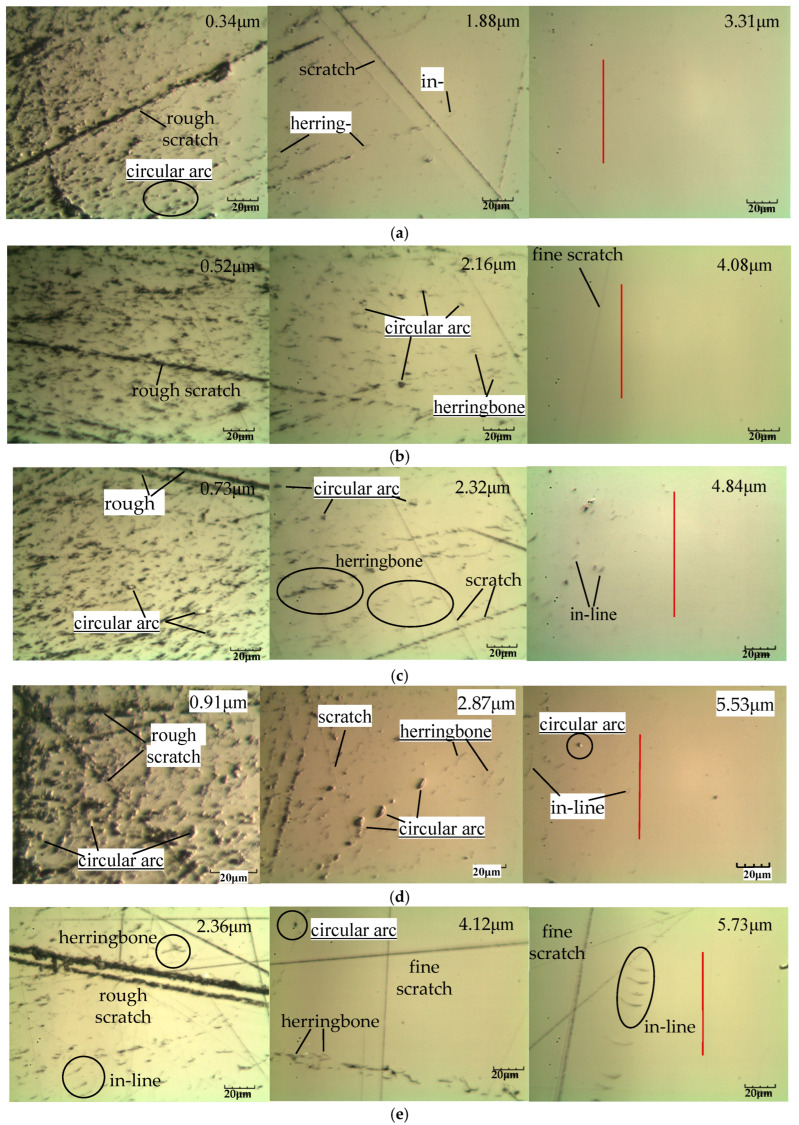
Subsurface damage images of wafers at different subsurface depths in each group: (**a**) 7 kPa; (**b**) 10.5 kPa; (**c**) 12.5 kPa; (**d**) 15 kPa; (**e**) 20 kPa.

The fixed lapping pad demonstrated strong holding force on abrasive grains, and most abrasive grains acted on the wafer surface in the form of two-body scribing. During scribing, the friction between abrasive grains and the wafer surface changed the stress distribution in the contact zone, generating peak tensile stress at the trailing edge of the abrasive grains, thus forming obvious “herringbone” cracks characterized by sharp indenter scribing and leaving fine scratches on the surface. Some abrasive grains fall off due to increased force after blunting, damaging the surrounding matrix, and a small number of arc-shaped cracks also appears on the wafer subsurface, as shown in Figure 8.

When the *P* decreased from 15 kPa to 7 kPa (by about 50%), the SSD decreased by approximately 40%, as shown in Figure 7b. This measurement was consistent with Lambropoulos’ theory that the median crack depth c was proportional to the 2/3 power of the load P. As shown in Figure 8a, almost all cracks under 7 kPa are “herringbone”, “in-line”, and shallow fine cracks. As shown in Figure 8d,e, coarse scratches and a small number of arc-shaped defects are present at 15 kPa and 20 kPa. Partial brittle removal occurred under both high-pressure conditions, resulting in a relatively stable SSD value. From the perspective of SSD, low pressure was conducive to reducing SSD and can effectively decrease the material removal amount and processing time in the subsequent process.

### 3.3. Analysis of Acoustic Emission Signal

**Figure 9:**
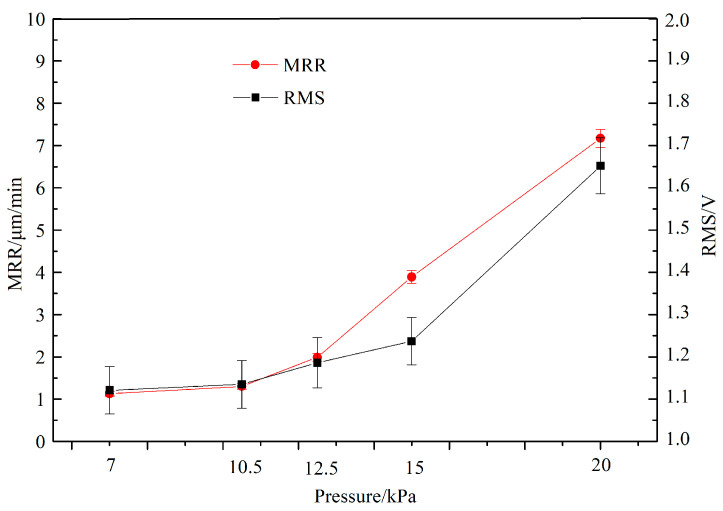
Relationship between the root mean square of the acoustic signal voltage and the MRR.

The AE mechanism during LN lapping may include plastic deformation, crack propagation, abrasive friction, abrasive wear, and slight adhesive wear. With the increase in polishing pressure, the cutting depth of single abrasive grains increased. The rise in normal and tangential forces excited stronger elastic waves, leading to an increase in RMS. The test results of RMS values are presented in Figure 8, showing that RMS rises with increasing P. As illustrated in Figure 9, RMS exhibits a linearly positive correlation with the MRR. The correlation coefficient obtained from data fitting is shown in Equation (4), with a correlation coefficient R = 0.97 (R^2^= 0.94), indicating a positive correlation:

(4) MRR=11.11RMS−10.97

At 7 kPa to 12.5 kPa, the cutting depth is small due to the elastic retreat of the polishing pad, and the material removal mechanism is dominated by plowing (fine scratches induced by plowing, as shown in Figure 6f and Figure 8a), resulting in a low RMS value. As the pressure increases, the RMS value raised slowly, and both the MRR and SSD presented an upward trend. This was mainly because the cutting depth gradually increased with rising pressure, and the material removal mode transformed from ductile removal to brittle removal with an increased proportion of brittle removal. Meanwhile, high load promotes deeper crack propagation, forming a deeper subsurface damage layer, as evidenced by arc-shaped defects with typical brittle removal characteristics in the subsurface at 15 kPa (Figure 8e). When the pressure reaches 20 kPa, brittle removal dominates and abrasive wear appeared. The increased friction on the contact surface after abrasive blunting caused an abnormal rise in RMS and the MRR nearly doubles, while SSD remained basically unchanged. Experiments indicated that RMS can serve as an online monitoring indicator for both the MRR and SSD during the polishing process. To achieve a high MRR and low SSD, the RMS should be controlled near the critical value of ductile–brittle transition. This not only ensured sufficient energy for efficient material removal, but also avoided deep subsurface damage caused by excessive energy input.

## 4. Conclusions

1. In lapping experiments, high pressure was beneficial to improving MRR, while low pressure was conducive to obtaining smooth surfaces, namely a low Ra value. The MRR increased continuously with the rise in pressure, whereas Ra increased slowly at first, then increased rapidly, and finally tended to stabilize as pressure increases.
2. Low pressure was conducive to reducing SSD, where material removal dominated by ductile mode and induced damages were mainly “in-line” and fine scratches. In contrast, material removal under high-pressure was predominantly brittle, resulting in the formation of herringbone cracks, arc-shaped cracks, and coarse scratches in the damage layer. From the perspective of damage control, a relatively low pressure was preferred on the premise of guaranteeing processing efficiency, which can effectively reduce the removal amount and processing time of subsequent processes.
3. RMS increased with rising pressure, and its growth rate was closely related to the material removal mechanism. The RMS value increased slowly with increasing pressure during ductile removal, continuously in the ductile–brittle transition region, and abnormally under brittle removal. RMS showed a linearly positive correlation with the MRR and grows synchronously with SSD in the brittle domain. The RMS value can be adopted as an online monitoring index for the MRR and SSD. Controlling RMS near the critical value of ductile–brittle transition is key to achieving high-efficiency and low-damage machining.
4. A comprehensive comparison of the five experimental groups showed that the total material removal under 7 kPa was close to the SSD of the workblank, which met the expected lapping removal capacity. Meanwhile, it introduced the minimum additional SSD and yielded a low Ra. To improve the efficiency of subsequent processes, the experimental parameters of this group were finally determined as the optimal thinning process: pressure of 7 kPa, head speed of 100 r/min, table speed of 80 r/min, slurry flow rate of 100 mL/min, eccentricity of 60 mm, a soft lapping pad matrix, abrasive mass fraction of 50 wt%, and lapping time of 5 min. After lapping, Ra was within 30 nm, the MRR exceeded 1 μm/min, and SSD was as low as 3.3 μm.

## Figures and Tables

**Table 1:** Additives and components in pad.

Pad Composition	Matrix	Abrasive	Others
Content	Hydrophilic unsaturated polyester resin 100/wt%	W28 Diamond 100/wt%	MgSO_4_ 15/wt% W10 SiC 30/wt%

**Table 2:** Lapping experimental parameters.

Speed /r/min	Eccentricity /mm	Slurry Flow Rate /mL/min	Lapping Time /min	Pad Matrix	Abrasive Mass Fraction /wt%
Head 100/ Table 80	60	100	5	Soft (E = 0.6 GPa, pendulum hardness 276 min^−1^)	50

Table 80 refers to the rotation speed of the “Table” being 80 r/min.

**Table 3:** Inclined surface polishing parameters.

Polishing Pad	Table Speed/r/min	Load	Slurry	Flow Rate/mL/min	Time/min
Polyurethane	50	Weights of 1100 g	90–100/nm Silica sol	15	30

## Data Availability

The original contributions presented in this study are included in the article. Further inquiries can be directed to the corresponding authors.

## Abbreviations

The following abbreviations are used in this manuscript:

AE	Acoustic emission signal
BBAAT	Flexible ball-end body-armor-like abrasive tool
CMP	Chemical mechanical polishing
CZT	Cadmium zinc telluride
FAP	Fixed abrasive polishing
LN	Lithium niobate
LT	Lithium tantalite
MRD	Material removal depth
MRR	Material removal rate
Ra	Surface roughness
RMS	Root mean square of acoustic emission signal
SSD	Subsurface damage depth
